# The *In Utero* Programming Effect of Increased Maternal Androgens and a Direct Fetal Intervention on Liver and Metabolic Function in Adult Sheep

**DOI:** 10.1371/journal.pone.0024877

**Published:** 2011-09-14

**Authors:** Kirsten Hogg, Charlotte Wood, Alan S. McNeilly, W. Colin Duncan

**Affiliations:** MRC Centre for Reproductive Health, The University of Edinburgh, Edinburgh, United Kingdom; Boston University, United States of America

## Abstract

Epigenetic changes in response to external stimuli are fast emerging as common underlying causes for the pre-disposition to adult disease. Prenatal androgenization is one such model that results in reproductive and metabolic features that are present in conditions such as polycystic ovary syndrome (PCOS). We examined the effect of prenatal androgens on liver function and metabolism of adult sheep. As non-alcoholic fatty liver disease is increased in PCOS we hypothesized that this, and other important liver pathways including metabolic function, insulin–like growth factor (IGF) and steroid receptivity, would be affected. Pregnant ewes received vehicle control (C; n = 5) or testosterone propionate (TP; n = 9) twice weekly (100 mg; i.m) from d62–102 (gestation 147 days). In a novel treatment paradigm, a second cohort received a direct C (n = 4) or TP (20 mg; n = 7) fetal injection at d62 and d82. In adults, maternal TP exposure resulted in increased insulin secretion to glucose load (*P*<0.05) and the histological presence of fatty liver (*P*<0.05) independent of central obesity. Additionally, hepatic androgen receptor (*AR*; *P*<0.05), glucocorticoid receptor (*GR*; *P*<0.05), UDP- glucose ceramide glucosyltransferase (*UGCG; P*<0.05) and *IGF1* (*P*<0.01) expression were upregulated. The direct fetal intervention (C and TP) led to early fatty liver changes in all animals without differential changes in insulin secretion. Furthermore, hepatic phosphoenolpyruvate carboxykinase (*PEPCK*) was up-regulated in the fetal controls (*P*<0.05) and this was opposed by fetal TP (*P*<0.05). Hepatic estrogen receptor (*ERα*; *P*<0.05) and mitogen activated protein kinase kinase 4 (*MAP2K4*; *P*<0.05) were increased following fetal TP exposure. Adult liver metabolism and signaling can be altered by early exposure to sex steroids implicating epigenetic regulation of metabolic disturbances that are common in PCOS.

## Introduction

An abnormal or altered fetal environment is emerging as a likely etiological factor for a host of adult diseases [1], [2]. The maternal environment can influence epigenetic processes in the placenta and fetus that program lasting developmental changes associated with cardiovascular disease, hypertension, obesity, type II diabetes, endocrine disruption and reproductive anomalies [2], [3], [4], all of which are prevalent in Westernized societies. Polycystic ovary syndrome (PCOS) is a common endocrine, ovarian and metabolic disorder in young women and is proposed to have early fetal origins [5], [6]. Animal models of PCOS have been established through investigation of the effects of increased male sex hormone during pregnancy. Prenatal androgenization of a female fetus results in the development of features characteristic of PCOS in non-human primates, rats and sheep [7], [8], [9], [10], [11]. Midgestation androgen exposure in sheep leads to polyfollicular ovaries, increased luteinizing hormone (LH), dysregulation of neuroendocrine feedback and ultimately disrupted estrous cycles in adult offspring [11], [12], [13]. Metabolic parameters such as hyperinsulinemia and insulin resistance are also reported [14], [15].

We hypothesized that the prenatal androgenization ovine model, leading to adult reproductive and metabolic defects, could inform us about early tissue-specific molecular changes associated with the PCOS-like phenotype, with particular focus on the liver. Women with PCOS are more likely to have abnormal levels of liver function determinants [16], [17] and non-alcoholic fatty liver disease (NAFLD) [18], [19], [20]. NAFLD occurs through liver damage, steatosis and in severe cases hepatic inflammation, and is associated with central obesity and insulin resistance [21]. Women with PCOS have biochemical features of hyperandrogenism [6], and androgens have a direct link with the development of central obesity and insulin resistance [22], [23]. Thus the metabolic features of PCOS involve a complex interplay between increased androgen concentrations, insulin signaling, central obesity and NAFLD [6], [24].

An assessment of NAFLD in young adult ewes exposed to increased androgen concentrations *in utero,* and investigation of the metabolic and synthetic function of the liver at a molecular level, was carried out. The relationship between these findings and changes in androgens, insulin resistance and central obesity was determined to provide novel insights into possible early hepatic alterations associated with a PCOS-like condition. We aimed to establish whether prenatal androgenization: 1) is associated with the development of NAFLD or clinical determinants of liver damage, 2) is linked to the development of central obesity and increased circulating androgen concentrations and, 3) results in a metabolic phenotype affecting glucose and insulin homeostasis. We investigated whether prenatal androgenization affected adult liver function by examining the molecular expression of: 4) hepatic steroid receptors, 5) the gluconeogenic enzyme phosphoenolpyruvate carboxykinase (PEPCK) and other genes involved in metabolic function, and 6) growth factor secretion by analyzing hepatic expression of insulin-like growth factor 1 (IGF1).

Finally, we introduced a novel direct fetal intervention to assess the effect of prenatal androgens on the adult hepatic phenotype avoiding interference from placental aromatization of testosterone [25] or the maternal effects of androgens. Young adult ewes were directly injected with depot testosterone at d62 and d82 of gestation and compared to those exposed to androgen from d62 to d102 using conventional maternal administration of testosterone. The timings of direct fetal injection were chosen to coincide with the period of increased maternal androgens encountered by the indirect treatment cohort.

Herein, we report the metabolic consequences of prenatal androgenization that include fatty liver and perturbed insulin signaling in young adult offspring, as well as intrinsic alterations in hepatic gene expression that may contribute to the overall metabolic phenotype observed in these animals. The direct treatment paradigm resulted in differential effects on hepatic outcome and insulin response compared to the indirect treatment some of which were also regulated by androgens, indicating programming by alternate pathways.

## Results

### Maternal prenatal androgenization is associated with subclinical fatty liver in adults that is not related to central obesity

**Figure 1:**
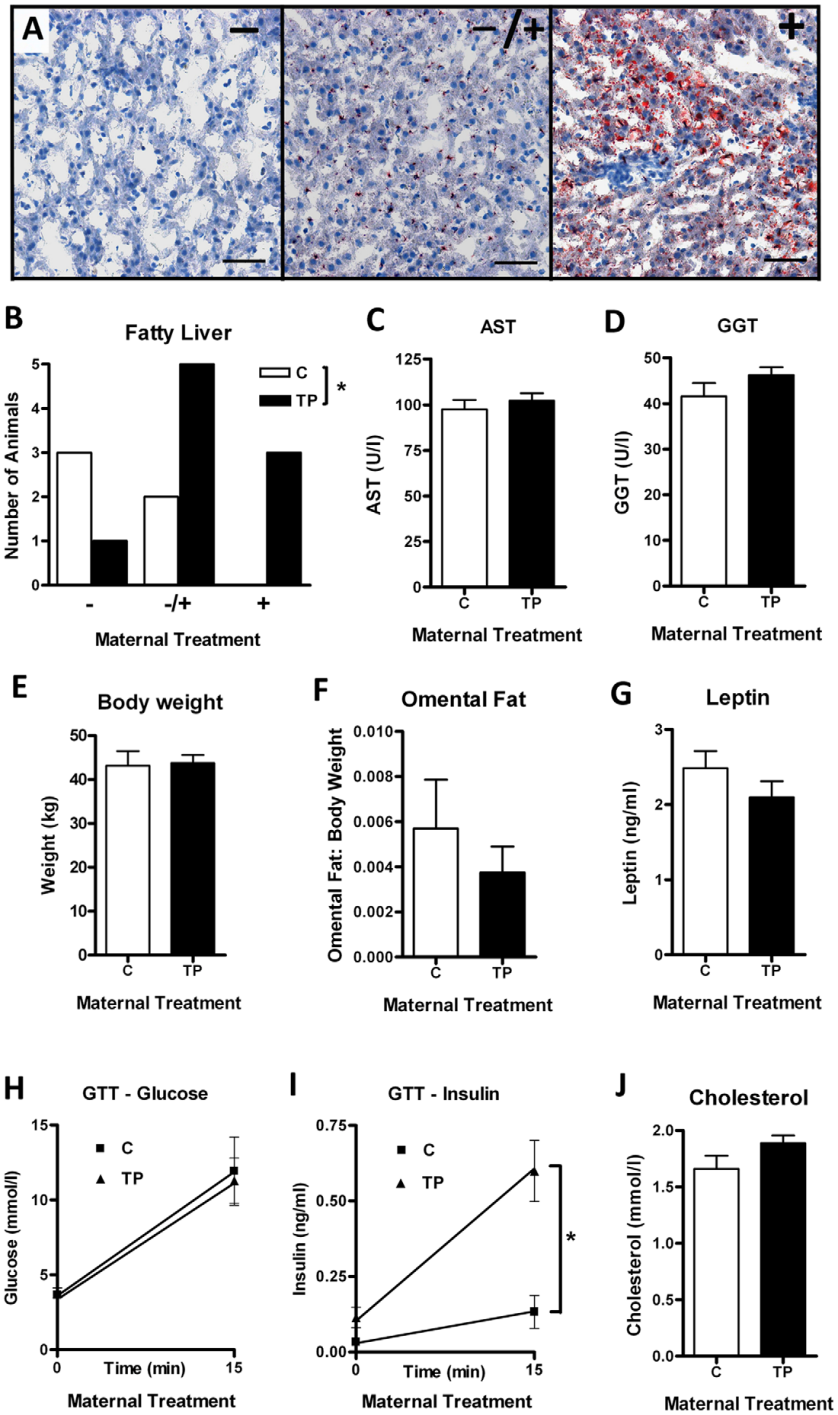
Effect of maternal prenatal androgenization on adult liver lipid content and other metabolic parameters. A) Representative oil red O histological stains for lipids in ovine adult liver sections illustrating negative (-), possible early (+/−) or positive (+) fatty liver (scale bars = 50 µm) and B) analysis of control (C; n = 5) and maternal TP (n = 9) showing increased fatty liver in the TP cohort. Maternal prenatal TP treatment had no effect on adult liver analytes including aspartate transaminase (AST; C) and gamma glutamyltransferase (GGT; D). There was no effect on body weight (E), weight of omental fat (F), plasma leptin (G) and cholesterol levels (J). H) Plasma glucose concentrations at basal levels and 15 min after i.v. glucose bolus showed no differences, however, after 15 min there were significantly higher plasma insulin concentrations in the TP group (I). Values are mean ± SEM and * = *P*<0.05.

To determine whether maternal prenatal androgenization is associated with the development of fatty liver in young adult sheep, liver tissue was examined for signs of lipid accumulation and plasma was analyzed for clinical features of liver damage. An oil red O histological lipid stain was performed on frozen liver sections and staining was classified into negative (-), possible early (+/−), or positive (+) fatty liver (Fig. 1A), as determined by the presence of red lipid globules. Androgenization of female fetuses by maternal testosterone propionate (TP) treatment from d62–102 of gestation resulted in the presence of fatty liver in offspring at 11 months of age (Fig. 1B; *P*<0.05). These early signs of liver damage were not however detectable at a clinical level as there was no change in serum determinants of liver function including circulating aspartate transaminase (AST; Fig. 1C), alanine transaminase (ALT; C = 19.4±2.8 U/l Vs TP = 16.6±1.1 U/l), alkaline phosphatase (ALP; C = 179.8±23.9 U/l Vs TP = 200.1±29.4 U/l) and gamma glutamyltransferase (GGT; Fig. 1D) concentrations. Whilst fat accumulation was present in the liver, prenatal testosterone exposure did not lead to increased body weight (Fig. 1E) or central obesity (omental fat; Fig. 1F) in adult sheep. These findings were consistent with the lack of change in circulating leptin concentrations (Fig. 1G).

### Insulin homeostasis in maternally treated adult offspring

The presence of fatty liver without central obesity in adults from androgenized mothers indicates early metabolic disruption that might be further manifested systemically. Glucose tolerance tests (GTTs) were carried out just prior to sacrifice to determine pancreatic response to glucose. Following bolus glucose administration, initial glucose dynamics between control and TP-exposed animals were not altered (Fig. 1H), however glucose-stimulated insulin secretion was significantly greater in the treatment group (Fig. 1I; *P*<0.05), suggesting a perturbed pancreatic response. Basal circulating insulin levels were higher in adults maternally exposed to TP (Fig. 1I), though this did not reach statistical significance (*P* = 0.071). Other metabolic parameters including plasma free fatty acids (FFA; C = 0.74±0.41 mmol/l Vs TP = 0.60±0.17 mmol/l), triglycerides (C = 0.3±0.07 mmol/l Vs TP = 0.28±0.02 mmol/l) and cholesterol (Fig. 1J) were not altered.

### Alterations in the expression of hepatic steroid receptors

**Figure 2:**
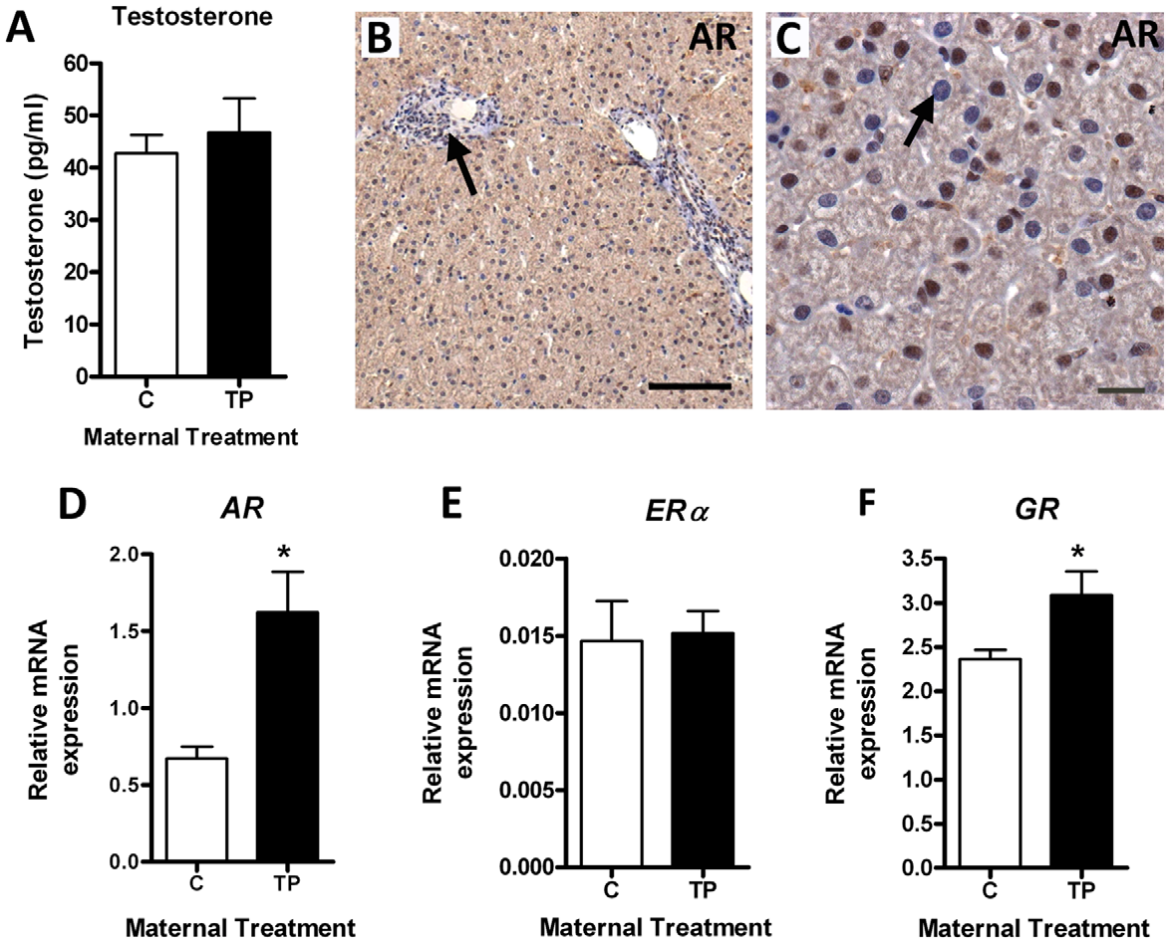
Effect of maternal prenatal androgenization on adult liver steroid receptor expression. A) There was no difference in adult plasma testosterone after maternal TP compared to controls. B–C) Immunohistochemistry showing androgen receptor (AR) localization in adult ovine liver sections (B, scale bar = 100 µm; arrow indicating staining in blood vessels and C, scale bar = 20 µm; arrow indicating negatively stained hepatocyte nucleus). Representative sections were selected from control animals. QRT-PCR showing the effect of maternal androgenization on hepatic *AR* (D), *ERα* (E) and *GR* (F) mRNA expression. Values represent mean ± SEM and * = *P*<0.05.

As subclinical fatty liver, and insulin resistance, can be associated with increased androgens we examined androgen concentrations and the potential of the adult liver to respond to androgen. There was no change in circulating testosterone concentrations in young adults after maternal TP exposure (Fig. 2A). Unfortunately, suitable assays for sex hormone binding globulin (SHBG) in the sheep were not available to measure free androgen index. However, mRNA analysis did not reveal significant changes in *SHBG* transcript abundance (C = 1.46±0.17 Vs TP = 2.08±0.32). Androgen receptor (AR) was found to be expressed in the nuclei and more weakly in the cytoplasm of hepatocytes across the parenchymal region (Fig. 2B), as well as in the nuclei of cells associated with portal veins (arrow; Fig. 2B). Interestingly, whilst a strong nuclear stain was observed in many hepatocytes, a large proportion of cell nuclei were also negative for AR (arrow; Fig. 2C). This pattern did not appear to be altered by prenatal androgens. QRT-PCR analysis revealed an upregulation of *AR* in the livers of prenatally TP-exposed adult sheep (Fig. 2D; *P*<0.05). There was no difference in expression of genomic estrogen receptors (*ERα*; Fig. 2E) but there was a small increase in the hepatic expression of glucocorticoid receptors (*GR*; Fig. 2F; *P*<0.05).

### Functional changes in the adult liver

**Figure 3:**
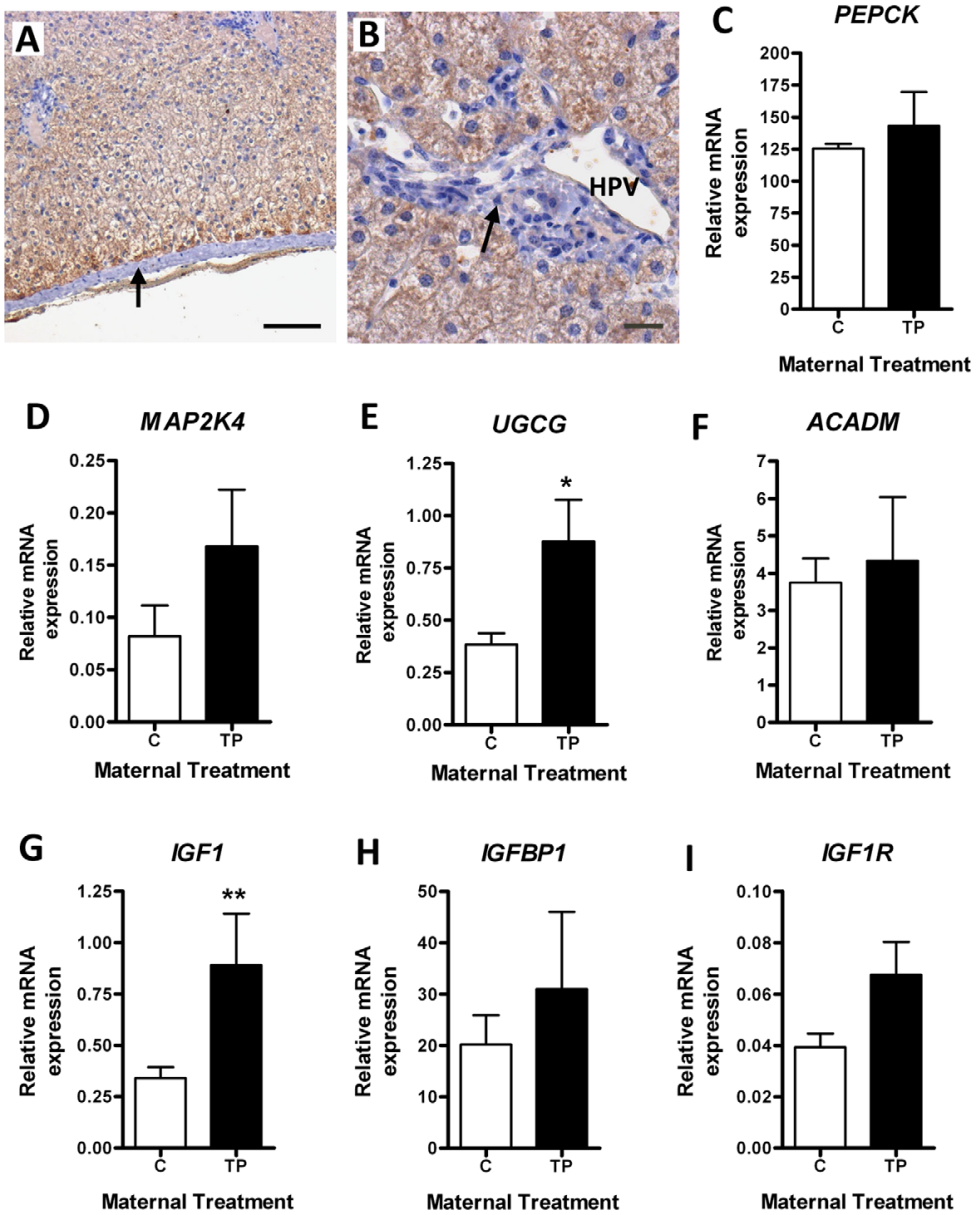
Effect of maternal prenatal androgenization on hepatic metabolic and signaling pathways. A-B) Immunohistochemistry for PEPCK (brown) in adult liver sections. A) Hepatocytes are stained while supporting tissue, such as the capsule (arrow) are not immunostained (scale bar = 100 µm). B) Higher power section of the more intensely stained hepatocytes around the hepatic portal vessels (HPV). Arrow indicates no staining associated with the non-hepatocytes in this region (scale bar = 20 µm). Representative sections were selected from control animals. QRT-PCR showing the effect of maternal androgenization on hepatic *PEPCK* (C), *MAP2K4* (D), *UGCG* (E), *ACADM* (F), *IGF1* (G), *IGFBP1* (H) and *IGF1R* (I) mRNA expression. Values represent mean ± SEM and * = *P*<0.05, ** = *P*<0.01.

To determine whether there were functional changes in the liver itself that may be associated with metabolic dysregulation we investigated the effect of prenatal androgens on hepatic gluconeogenesis by examining liver PEPCK expression. Immunolocalization of PEPCK (Fig. 3A) revealed widespread protein expression throughout parenchymal liver tissue with positive staining visualized within the cytoplasm and membrane of hepatocytes particularly in the peri-portal region. The liver capsule, comprising connective tissue, was negative for PEPCK (arrow; Fig. 3A) as were the non-hepatocyte cells surrounding the hepatic portal vein (HPV; arrow; Fig. 3B). However, there were no changes in *PEPCK* expression in prenatally TP-treated animals (Fig. 3C). Other candidate genes involved in metabolic function in the liver, including mitogen activated protein kinase kinase 4 (*MAP2K4*), UDP- glucose ceramide glucosyltransferase (*UGCG*) and acyl-coenzyme A dehydrogenase (*ACADM*) were studied. Maternal androgenization resulted in a 2-fold increase in hepatic *MAP2K4* (Fig. 3D; trend) and upregulation of *UGCG* (Fig. 3E; *P*<0.05), whereas *ACADM* mRNA was not altered by treatment (Fig. 3F). There were also transcriptional differences in the components of hepatic IGF system in prenatal TP-exposed adults, in which *IGF1* was upregulated ∼3-fold (Fig. 3G; *P*<0.01) without any significant change in *IGFBP1* expression (Fig. 3H) or *IGF1R* (Fig. 3I).

### Fetal exposure to testosterone per se might not be responsible for the subclinical fatty liver change

**Figure 4:**
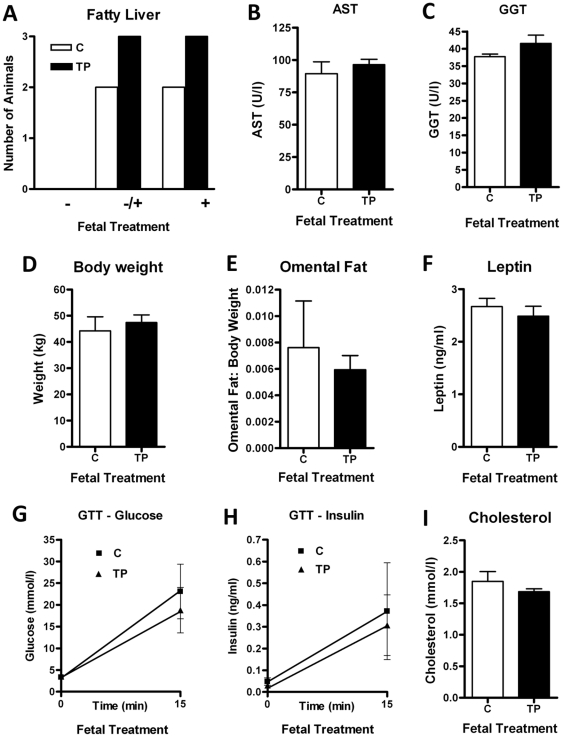
Effect of direct fetal androgenization on metabolic parameters in adult offspring. A) There was no difference on the development of fatty liver between control (C; n = 4) and TP (n = 7) administration. Fetal prenatal TP treatment did not affect adult liver analytes including AST (B) and GGT (C). There was no effect on body weight (D), weight of omental fat (E) and plasma leptin (F) and cholesterol levels (I). Plasma glucose (G) and insulin (H) concentrations at basal levels and 15 min after i.v. glucose bolus showed no differences. Values are mean ± SEM.

To assess the programming effects of prenatal androgenization without maternal effects or the contribution of placental metabolism of TP, metabolic and liver outcomes were also investigated in adult offspring directly treated with depot TP or vehicle control by ultrasound-guided injection at d62 and d82 of gestation. There was no difference in the proportion of adult animals histologically categorized for fatty liver between TP treatment and control (Fig. 4A). In fact, and unexpectedly, all animals showed signs of possible, or positive lipid accumulation (Fig. 4A) and the prevalence of fatty liver was similar to maternal TP exposed animals rather than maternal control injection animals. Nevertheless, like the maternal treated cohorts, liver function determinants including serum AST (Fig. 4B), ALT (C = 17.5±1.7 U/l Vs TP = 15.3±1.7 U/l), ALP (C = 247.3±30.3 U/l Vs TP = 197.6±19.7 U/l) and GGT (Fig. 4C) were not altered by direct fetal androgenization. In addition, there were no differences in body weight (Fig. 4D), central obesity (omental fat; Fig. 4E) or circulating leptin concentrations (Fig. 4F).

### Insulin homeostasis in direct fetally treated adult offspring

There were no significant differences between direct fetal control and TP animals when glucose (Fig. 4G) and insulin (Fig. 4H) levels were determined by GTT in adult animals. This suggests that fetal TP exposure does not directly affect insulin homeostasis. However, like the changes in hepatic lipid content, the direct fetal intervention alone also affected insulin secretion in response to glucose load. Although baseline glucose was similar in the fetal and maternal treatment groups, 15 min after a glucose bolus the serum glucose concentrations in the fetal treatment group were approximately double those of the maternal treatment group (23.1±6.3 mmol/l Vs 11.9±2.3 mmol/l, respectively; *P* = 0.06). In addition, the fetal control insulin concentrations after 15 min were mid-way between the maternal control and TP treatments (Fig. 4H, Fig. 1I). Like the fatty liver findings, this implies that the fetal injection *per se* may result in a metabolic phenotype that is not changed by the presence of testosterone and may affect glucose and insulin regulation. There were no changes in circulating FFA (C = 0.52±0.03 mmol/l Vs TP = 0.50±0.04 mmol/l), triglyceride (C = 0.32±0.06 mmol/l Vs TP = 0.20±0.04 mmol/l) or cholesterol concentrations (Fig. 4I).

### Alterations in the expression of hepatic steroid receptors

**Figure 5:**
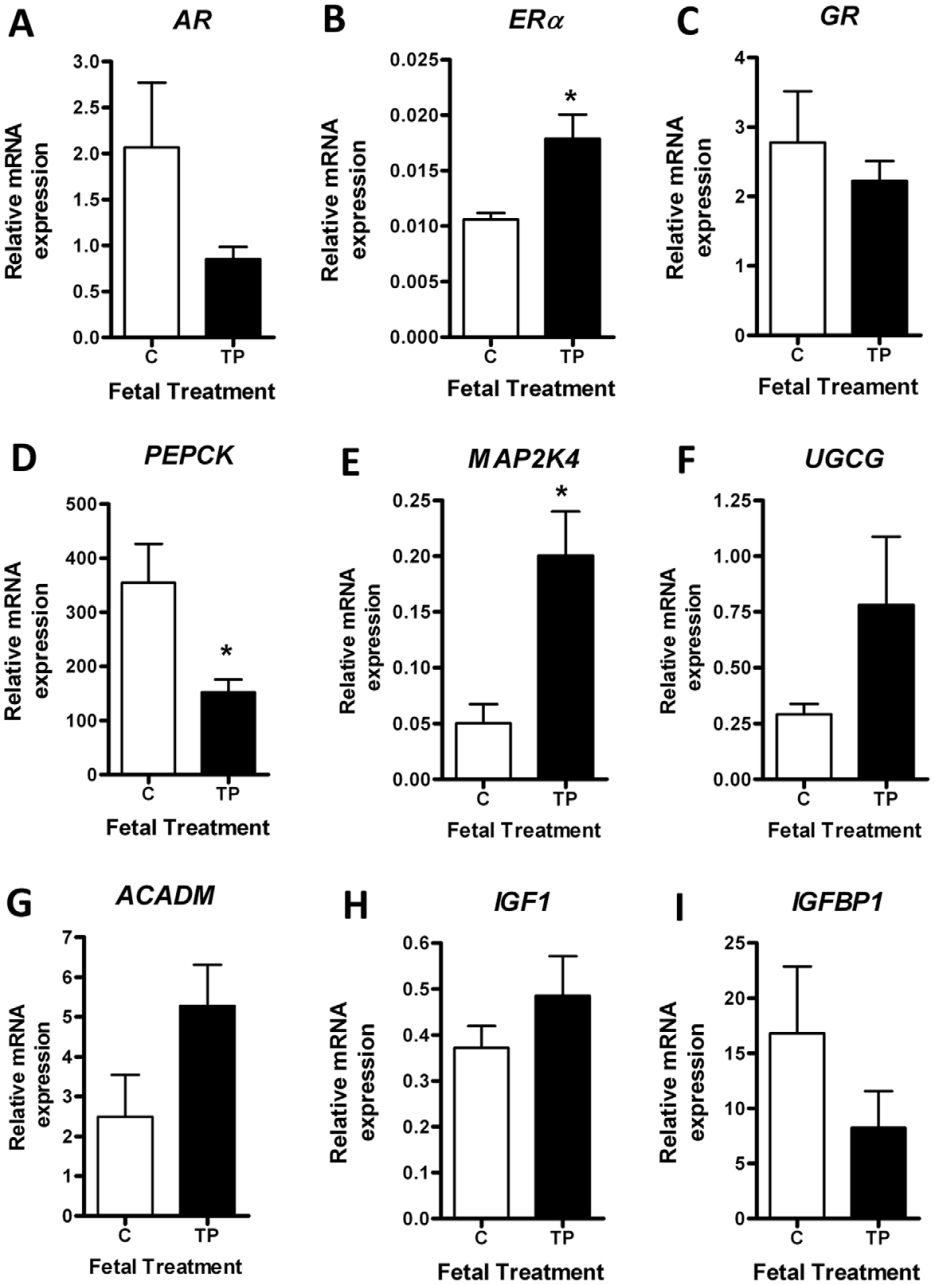
Effect of direct fetal androgenization on molecular liver function in adult offspring. QRT-PCR showing the effect of prenatal fetal androgenization on hepatic *AR* (A), *ERα* (B), *GR* (C), *PEPCK* (D), *MAP2K4* (E), *UGCG* (F), *ACADM* (G), *IGF1* (H) and *IGFBP1* (I) mRNA expression. Values represent mean ± SEM and * = *P*<0.05.

There were no differences in adult serum androgen concentrations after direct fetal treatment with TP (C = 42.8±13.1 pg/ml Vs TP = 42.6±5.51 pg/ml). *SHBG* mRNA expression was also unaltered (C = 1.27±0.15 Vs TP = 1.12±0.16). Unlike the maternal TP-treatment cohort hepatic *AR* gene expression was not increased by fetal TP exposure (Fig. 5A), in fact showing a tendency to be reduced. However, it was noted that *AR* mRNA expression in fetal controls was in fact 3 times that of maternal controls (2.1±0.70 Vs 0.67±0.12, respectively; *P* = 0.11). Also, in contrast to the maternal treatment, direct fetal TP treatment led to the upregulation of *ERα* gene expression in adult livers (Fig. 5B; *P*<0.05). No change in hepatic *GR* mRNA expression was observed in these animals (Fig. 5C).

### Functional changes in the adult liver

Adult liver *PEPCK* mRNA expression was downregulated following direct fetal TP exposure (Fig. 5D; *P*<0.05), in contrast to findings in the maternal androgenized cohort. However, the phenotypic effect of *in utero* injection was once more highlighted by a significant upregulation of *PEPCK* mRNA levels in fetal control compared to maternal control (354.4±71.6 Vs 125.6±5.2, respectively; *P*<0.05). Similarly to the findings in the maternal TP cohort hepatic metabolic genes were increased in response to fetal androgenization. *MAP2K2* was upregulated 4-fold (Fig. 5E; *P*<0.05) and both *UGCG* (Fig. 5F) and *ACADM* (Fig. 5G) tended to increase so that the average mean transcript abundance in TP-exposed animals was twice that of controls. In contrast to maternal treatment, direct prenatal androgen exposure did not lead to significant alterations in the hepatic IGF system. There were no changes in *IGF1* (Fig. 5H), *IGFBP1* (Fig. 5I), or *IGF1R* (C = 0.059±0.014 Vs TP = 0.051±0.017) gene expression.

## Discussion

Epigenetic programming of reproductive and metabolic function in the adult through maternal androgenization of the fetus is well documented [7], [10], [11], [13], [15], and has been likened to the features common to PCOS in women [5], . In an ovine model of midgestation androgenization, we investigate the metabolic and hepatic consequences of both indirect maternal and direct fetal androgenization in young adult sheep. The fetal androgenization model is novel with direct injection of the fetus with depot TP, and thus these effects can be compared with the fetal programming effect of increased testosterone in the mother. As such this treatment paradigm was performed in parallel to ewes receiving maternal TP over a comparative gestational period to delineate between the maternal impact of TP and any effects of placental aromatization of androgens [25], with those of direct TP effects in the fetus.

Fatty liver has not been previously reported in animal models of PCOS, however, NAFLD incidence is increased in women with PCOS and is associated with elevated aminotransferases, hyperandrogenemia and central obesity [18], [19], [20], [26], [27]. Maternal androgenization resulted in subclinical hepatic steatosis in young sheep, which was not associated with increased central (omental) fat, basal hyperinsulinemia or increased peripheral testosterone concentrations. In clinical terms, liver damage was not discernable as standard liver function tests, including ALT, AST, ALP and GGT, remained normal. This model may therefore present a pre-liver phenotype that is independent of the metabolic and hormonal features of PCOS and that could develop further to a detrimental metabolic consequence of prenatal androgenization, similarly to the progression of the reproductive phenotype as the animals age [8], [28].

As these sheep showed evidence of pancreatic dysregulation it remains unclear whether there is a temporal relationship or association between the development of fatty liver and increased insulin concentrations [29]. Hyperinsulinemia is a common feature of PCOS that results from a pancreatic β-cell defect [30], and can culminate in peripheral insulin resistance as the phenotype develops in both non-obese and obese patients [24], [31]. During intravenous GTT, insulin response to glucose load was significantly increased in maternally androgenized animals, implying a defect in pancreatic insulin secretion. While not a direct measure of insulin sensitivity, these findings may suggest reduced responsiveness of peripheral tissues to insulin and subsequently an enhanced requirement for insulin secretion. These animals did not appear to be glucose intolerant at this stage since glucose clearance was unaffected. Previous studies have shown that prenatal TP from d30–90 and/or d60–90 lead to reduced insulin sensitivity in 5 week [15] and 11 week [14] old lambs and that the critical developmental programming period for insulin signaling is during d60–90 [14]. Additionally, these studies reveal the parallel effects of prenatal TP and non-aromatizable dehydrotestosterone (DHT) indicating an androgenic mode of programming [14]. For technical reasons we were unable to conduct a euglycemic clamp to directly measure insulin sensitivity, however, our findings further underline intrinsic changes that occur prior to alterations in body weight, fat distribution or circulating androgen concentrations.

In parallel to investigating the programming effects of increased androgens in the mother we also attempted to study the effects directly in the fetus and analyze the same metabolic and hepatic parameters as in the traditional model. Direct fetal exposure to TP during the same treatment window did not alter the proportion of young adults that had hepatic lipid accumulation, but remarkably all animals, including the control cohort, presented physical signs of fatty liver. No animals in this model presented with altered central obesity, circulating androgen or insulin concentrations. Interestingly, during GTT, insulin secretion in response to glucose was raised in both treatment groups so that the mean value fell mid-way between maternal control and TP groups. Epigenetic programming arising as a direct result of this fetal treatment intervention is of interest and might arise through maternal anesthesia, the injection itself or the oil used the dilute the TP and could therefore be a consequence of stress or inflammatory induced pathways. While the underlying mechanisms leading to these findings will require further interrogation, it appears that at higher concentrations [32], for at least as long, of fetal androgens did not program additional fatty liver changes. This is important as it could imply that the changes observed in the maternal treated cohort may not arise through the direct actions of androgens *per se* and confirms that some features of prenatal androgenization animal models of PCOS are not directly related to androgen exposure [33], [34], [35].

Since these animals showed early signs of liver damage, a comprehensive molecular analysis was carried out in these livers to identify mechanistic pathways that might be altered. These findings are discussed below and are summarized in Table 1 for clarity. The finding of altered adult liver lipid composition in both models prompted the investigation of hepatic modulators of metabolism and glucose homeostasis. A candidate for study was PEPCK, which in the liver catalyzes the conversion of oxaloacetate into phosphoenolpyruvate and is the rate limiting step of gluconeogenesis [36]. Gluconeogenesis is an essential hepatic process to maintain normal blood glucose concentrations. *PEPCK* undergoes tight transcriptional control and can be regulated by hormones, including insulin that has an inhibitory role and glucagon and glucocorticoids that stimulate its expression [37].

**Table 1:** Summary of the effects of differing fetal programming models on hepatic gene expression in the adult ewe.

Gene	Maternal TP Effect	Fetal TP Effect	Fetal Injection Effect
*AR*	↑*	↓	↑
*ERα*	-	↑*	-
*GR*	↑*	-	-
*PEPCK*	-	↓*	↑*
*MAP2K2*	↑	↑*	-
*UGCG*	↑*	↑	-
*ACADM*	-	↑	-
*IGF1*	↑*	-	-
*IGFBP1*	-	↓	-
*IGF1R*	↑	-	-

Upward arrows (↑) represent an increase and downward arrows (↓) represent a decrease in mean gene expression in TP cohorts compared to controls. The fetal injection column is compared to the maternal controls. Asterisks (*) indicate significantly altered gene expression. Dashes (-) represent no change.

Prenatal androgenization of the mother had no effect on hepatic *PEPCK* expression in the adult offspring. However, direct fetal treatment led to differential changes in *PEPCK* gene expression with a significant upregulation in fetal control compared to maternal control animals, but a downregulation in fetal TP compared to fetal control cohorts. In this instance the fetal injection phenotype was altered by direct exposure to androgens. PEPCK has been linked to the development of fatty liver in mice. Over-expression of *PEPCK* leads to a type II diabetic phenotype [38], however liver-specific knock-out does not affect blood glucose but does result in the development of fatty liver [39], suggesting a role in liver lipid metabolism distinct from gluconeogenesis [40], [41]. The role and regulation of liver *PEPCK* after fetal programming is complex and requires further work but it can be altered by fetal injection and modified by androgen exposure in the fetus.

In addition to *PEPCK*, other gene candidates that have roles in hepatic metabolic processes including *MAP2K4* (ceramide signaling), *UGCG* (ceramide metabolism) and *ACADM* (lipid metabolism) were studied. These genes were selected as they are known to be positively associated with fatty liver in human subjects [42]. MAP2K4 activates other MAP kinases to illicit downstream signaling in response to stress, pro-inflammatory cues or mitogenic stimuli and is essential for liver development in mice [43]. We found a trend for increased *MAP2K4* gene in livers from maternal androgenized offspring, and a 4-fold up-regulation of hepatic *MAP2K4* in animals that were directly androgenized. The enzyme UGCG catalyzes the initial glycosylation step of the synthesis of glycosphingolipids, which are important cell membrane components with varying cellular roles [44]. In our models, an upregulation of *UGCG* observed in adult livers was programmed by maternal androgens, and increased fetal androgens also affected this gene expression in a similar trend. During lipid metabolism, the first step of β-oxidation of fatty acids is catalyzed by ACADM. In addition to being associated with NAFLD in gene studies, increased serum β-hydroxybutyrate concentrations, as an indirect measure of fatty acid oxidation, are present in patients with fatty liver [45], [46]. While not affected by maternal androgenization, *ACADM* was also altered in an upward trend in those animals directly receiving prenatal androgens. Unlike *PEPCK*, these genes were not affected by injection paradigm and appear to be upregulated as a response to androgens. These findings indicate intrinsic molecular alterations occurring in the liver prior to the clinical manifestation of liver damage.

Given that PCOS is a reproductive and metabolic disorder, factors produced by the liver that could affect ovarian function as well as metabolism, namely IGF1 and its signaling system, were also studied. IGFBP1 sequesters IGF1 reducing bioavailability [47] and an increased IGF1:IGFBP1 ratio [48] and reduced IGFBP1 [49] in serum is associated with obesity, hyperinsulinemia and insulin resistance in human studies and in women with PCOS [50], [51]. Hepatic *IGF1* mRNA was significantly increased in maternal androgenized offspring so that alterations in the hepatic expression of IGF1 could be involved in the phenotype associated with ovine prenatal androgenization [13], [14], [15]. Conversely, direct androgenization of the fetus did not affect adult liver expression of *IGF1*. It is however likely that steroids have a role in the modulation of liver IGF1 expression. The human *IGF1* gene is known to contain androgen responsive elements in the upstream promoter region that bind AR and stimulate *IGF1* transcription [52]. Although circulating testosterone concentrations were not altered in these sheep, liver *AR* was upregulated after maternal androgen exposure and therefore these livers may be more responsive to androgens. Interestingly, *AR* gene expression in controls was also increased as a consequence of fetal injection paradigm and could therefore also enhance the receptivity of these livers to androgens if mirrored by protein expression. Unfortunately, due to the recognized technical limitations of Western blotting for IGF1 or AR in ovine tissues, we were unable to convincingly and robustly analyze expression at the protein level.

 In addition to changes in hepatic *AR* gene expression other alterations in hormone receptor expression were observed. These included the upregulation of *GR* following maternal androgenization, and differentially, the increase in *ERα* expression following fetal exposure to androgens. It is not known what the significance of these changes in potential hepatic receptivity to these steroid hormones is, but illustrates further intrinsic perturbations in liver function and signaling as a consequence of prenatal androgenization.

In an ovine model of prenatal maternal androgenization, we report the occurrence of hepatic steatosis, alterations in hepatic metabolic gene expression and perturbations in insulin secretion in young adults. These changes are independent of central obesity and hyperandrogenemia and may be the culmination of disturbances to hepatic metabolic homeostasis predisposed by exposure to *in utero* factors, including androgens, estrogens and/or stress. Direct injection of a midgestation fetus results in the universal development of fatty liver which suggests that some effects are not entirely attributable to programming by TP. Nevertheless, alterations in hepatic metabolic gene expression, as a consequence of direct fetal exposure, did mirror that of maternal androgen exposure. In a prenatal androgenization ovine model of PCOS, these data suggest that: 1) aspects of metabolic disturbance that occur in women with PCOS may be programmed before birth and are independent of secondary metabolic and/or hormonal modulators, and 2) androgens *per se* may not be directly responsible for the epigenetic programming of all PCOS-like features reported in offspring of androgenized mothers.

## Materials and Methods

### Ethics Statement

These studies were approved by the UK Home Office and were conducted under an approved Project Licence (PIL 60/3744) following review by the University of Edinburgh Animal Research Ethics Committee.

### Reagents

All reagents and chemicals were obtained from Sigma-Aldrich (Poole, UK), unless otherwise stated.

### Animal treatments and tissue collection

Scottish Greyface ewes were mated with Texel rams and singleton, twin and triplet pregnancies were evenly divided between treatment and control groups. Triplet pregnancies were not included in the fetal injection cohort. In the maternal injection cohort, pregnant ewes were treated intramuscularly twice weekly with 1 ml vehicle control (C) or 100 mg TP (AMS Biotechnology Europe Ltd, Abingdon, UK), in vegetable oil, from d62–102 of gestation (term is 147 days). In the fetal injection cohort, mothers were anesthetized prior to fetal injection by initial sedation using 10 mg Xylazine (i.m.; Rompun; Bayor Plc Animal Health Division, Berkshire, UK), followed by 2.0 mg/kg Ketamine (i.v.; Keteset; Fort Dodge Animal Health, Southampton, UK). Ewes were additionally administered a post-operative dose of antibiotics (1 ml/25 kg; i.m.; Streptacare; Animalcare Ltd., York, UK). Under sterile surgical conditions fetuses were injected at d62 and d82 of gestation into the flank once with 0.2 ml vehicle C or 20 mg TP using a 20 G Quincke spinal needle (BD Biosciences, Oxford, UK) under ultrasound guidance. The fetal injections were timed to commence on the same day as maternal injection and since we have previously shown that this TP depot is long-lasting [32], two injections 20 days apart were selected to best mirror the length of fetal exposure through maternal injections. Lambs were weaned at 3 months and fed hay or grass *ad libitum* until 11 months of age.

Adult female offspring numbers from maternal and fetal treatment groups were C = 5 and 4, and TP = 9 and 7, respectively. Animals were fasted and a GTT performed on the day of sacrifice with bolus glucose (500 mg/ml in 20 ml) administered intravenously following collection of a peripheral basal blood sample. Blood was re-sampled after 15 min and the animal immediately euthanized and tissue collected. Equivalent liver samples were snap frozen and stored at −80 C, or embedded in cassettes containing OCT compound (VWR International, Poole, UK) and snap frozen, or stored in Bouins fixative for 24 h, transferred to 70% ethanol and embedded in paraffin wax. In addition, the omental fat was removed and weighed. Blood was decanted into a heparinized test-tube for hormonal/metabolite measurements or S-Monovettes containing sodium fluoride combined with anti-coagulant (Sarstedt Ltd., Nümbrecht, Germany) for glucose measurement. Tubes were centrifuged at 3000 rpm for 15 min at 4 C and the plasma collected and stored at −20 C.

### Measurement of glucose and insulin

Glucose concentrations were measured with a colorimetric glucose assay kit (Alpha Laboratories Ltd., Eastleigh, UK), using a Cobas Fara centrifugal analyzer (Roche Diagnostics Ltd., Welwyn Garden City, UK). Assay sensitivity was 0.2 mmol/l and intra- and inter-assay coefficient of variation (CVs) were <2% and <3%, respectively. Insulin was measured using an ovine insulin ELISA kit (80-INSOV-E01, ALPCO Diagnostics, Salem, NH, USA) as per the manufacturer's instructions. Absorbance was measured colorimetrically on a ThermoMax Microplate Reader (Molecular Devices, CA, USA) at 450 nm with a reference wavelength of 650 nm. A standard curve comprising a cubic spline fit was generated and insulin concentrations calculated using SoftMax Pro software (Molecular Devices). Assay sensitivity was 5.0 pg/ml and intra- and inter-assay CVs were <5% and <6%, respectively.

### Measurement of other plasma analytes

To measure the concentration of cholesterol, triglycerides, FFA, ALT, AST, ALP and GGT, commercial assay kits were employed (Alpha Laboratories Ltd., Eastleigh, UK) as per the manufacturer's protocols, using a Cobas Fara centrifugal analyzer (Roche Diagnostics Ltd.) to obtain analyte concentration. Assay sensitivities for cholesterol, FFA, triglycerides, ALT, AST, ALP and GGT were 0.1 mmol/l, 0.05 mmol/l, 0.02 mmol/l, 4 U//l, 6 U/l, 20 U/l and 5 U/l, respectively, and the overall CVs were all <3%. Serum leptin concentrations were obtained using a Multi-Species Leptin radioimmunoassay kit with an antibody against human leptin (XL-85K, Millipore, Missouri, USA). Assay sensitivity was 1.0 ng/ml and intra- and inter-assay CVs were <4% and <7%, respectively. Serum testosterone concentrations were measured using an in-house radioimmunoassay method described previously [53], [54], where the intra- and inter-assay CVs were <10% and assay sensitivity 12 pg/ml.

### Oil Red O Lipid Staining and Analysis

Frozen 5 µm sections from OCT embedded liver samples were cut (Leica Cryostat Microtome 1900, Leica Biosystems Nussloch GmbH, Heidelberger, Germany) at −20 C, mounted onto glass slides and air dried. Tissue was fixed in 10% formalin for 10 min, rinsed in distilled water (dH_2_0) and air dried before a further rinse in 60% isopropanol. Sections were stained with Oil Red O dissolved in 60% isopropanol for 15 min at room temperature and rinsed in 60% isopropanol followed by washing in dH_2_0. Tissue was counterstained in hematoxylin, washed thoroughly in dH_2_0 and mounted in Aqua-Mount media (Thermo Fisher Scientific, Loughborough, UK). Unsaturated hydrophobic lipids were identified microscopically by the presence of a red stain and was subsequently quantified blindly by two independent investigators by classification into negative (-), possible early (−/+) and positive (+) fatty liver categories by comparison to pre-agreed reference sections.

### Quantitative (q)RT-PCR

RNA was extracted from a whole frozen liver sample using the TRI reagent method. Briefly, tissue was homogenized in 1 ml TRI reagent using a Qiagen TissueLyser (Qiagen Ltd., West Sussex, UK), rested for 5 min and phase separated with the addition of 0.2 ml chloroform and vigorous shaking for 15 sec. Samples were incubated for 5 min at room temperature and centrifuged at 12,000 g at 4 C for 20 min. The upper aqueous phase containing RNA was collected, mixed with 0.5 ml isopropanol, and incubated for 10 min at room temperature. Samples were centrifuged at 12,000 g at 4 C for 15 min and the RNA pellet washed in 1 ml 75% ethanol prior to centrifugation at 7,500 g and 4 C for a further 10 min. The pellet was subsequently air dried before elution in 50 µL nuclease-free water and stored at −80 C. RNA concentration and purity were measured using a NanoDrop 1000 spectrophotometer (Thermo Fisher Scientific) and cDNA was synthesized from 200 ng total RNA using the High Capacity cDNA reverse transcription kit (Applied BioSystems, CA, USA) and stored at −20 C.

Forward and reverse primers were designed for qRT-PCR (Table 2) using Primer3 Input v0.4 software [55]. Conventional PCR and PCR-product DNA sequencing was performed for validation of authenticity of the gene product in the sheep. Primer efficiency and suitability for SYBR Green qRT-PCR was further validated through generation of standard curves in qRT-PCR reactions. A 10 µL final reaction volume was prepared with Power SYBR Green PCR Master Mix (1X; Applied BioSystems), pre-diluted primer sets (0.5 µM), cDNA (1 µL) and nuclease-free water, with all reactions carried out in duplicate. The thermal cycling program included a denaturing step (95 C for 10 min), combined annealing and extension step (95 C for 15 sec, 60 C for 1 min [x40]), and a final dissociation step (95 C, 60 C and 95 C for 15 sec each). Negative controls per gene consisted of the use of cDNA prepared minus reverse transcriptase, and omission of cDNA from the qRT-PCR reaction. The expression of the unknown target gene relative to glyceraldehyde 3-phosphate dehydrogenase (*GAPDH)* as an internal control was quantified using the ΔCt method.

**Table 2:** Forward and reverse primer sequences, product sizes and accession numbers for genes analyzed by qRT-PCR. bp: base pairs.

Gene (accession)	Nucleotide Sequence (5′–3′)	Product size (bp)
*AR* (XM_001253942)		
Forward	GCCCATCTTTCTGAATGTCC	233
Reverse	CAAACACCATAAGCCCCATC	
*ERα* (NM_001001443)		
Forward	GAATCTGCCAAGGAGACTCG	187
Reverse	CCTGACAGCTCTTCCTCCTG	
*GR* (NM_001114186)		
Forward	AAGTCATTGAACCCGAGGTG	207
Reverse	ATGCCATGAGGAACATCCAT	
*MAP2K4* (NM_001099038)		
Forward	CAGCATTGAGTCGTCAGGAA	234
Reverse	TCACTGCTCCGCATCACTAC	
*UGCG* (NM_001076850)		
Forward	GGAGGTTTGCAATGTCCACT	157
Reverse	CTGGCAACAAAGCATTCTGA	
*ACADM* (NM_001075235)		
Forward	AACCTGTAGCAGGCTCTGATG	209
Reverse	TACTCCTGGGGTGTCTGCTT	
*IGF1* (NM_001009774)		
Forward	CATCCTCCTCGCATCTCTTC	239
Reverse	CTCCAGCCTCCTCAGATCAC	
*IGF1R* (XM_002696504)		
Forward	CCAAAACCGAAGCTGAGAAG	199
Reverse	TCCGGGTCTGTGATGTTGTA	
*IGFBP1* (NM_001145177)		
Forward	TCCCCAGAGAGCTCAGAGAT	207
Reverse	CTGCTCCCTGGCTAATCTGT	
*PEPCK* (NM_174737)		
Forward	AAAGAGATACGGTGCCCATC	178
Reverse	ATGCCAATCTTGGACAGAGG	
*SHBG* (NM_001098858)		
Forward	ACTTGGGATCCAGAGGGAGT	208
Reverse	CCACCCTGAGTAGCAAGGAA	
*GAPDH* (NM_001034034)		
Forward	GGCGTGAACCACGAGAAGTATAA	229
Reverse	AAGCAGGGATGATGTTCTGG	

### Immunohistochemistry

Immunohistochemistry was performed on 5 µm liver sections using AR and PEPCK antibodies. Tissue sections were dewaxed and rehydrated prior to antigen retrieval by pressure cooking (0.01 M sodium citrate buffer pH 6.0) for 5 min. Sections were washed (2×5 min) in phosphate buffered saline (PBS), incubated in 3% H_2_O_2_ for 10 min, followed by further PBS washes (2×5 min). Endogenous avidin/biotin sites were blocked to prevent non-specific antibody binding using the Avidin/Biotin Blocking Kit as per the manufacturer's instructions (Vector Laboratories, Peterborough, UK). Sections were incubated with blocking serum; 20% normal goat serum/5% bovine serum albumin/PBS, for 1 h. Primary antibodies; AR (1∶100, N20 rabbit polyclonal rabbit sc-816, Santa Cruz Biotechnology Inc., Santa Cruz, USA) or PEPCK (1∶100, H300 rabbit polyclonal sc-32879, Santa Cruz Biotechnology Inc.) were diluted in the blocking serum and applied to tissue overnight at 4 C.

Tissue sections were washed in PBST (PBS +1% tween; 2×5 min) before incubation with a biotinylated goat anti-rabbit IgG (1∶500, Dako, Glostrup, Denmark). After 2×5 min PBS washes, Vectastain ABC Elite tertiary complex (PK-1600 Series, Vector Laboratories) was applied for 1 h, and the tissue washed 2×5 min in PBS. Staining was visualized colorimetrically by application of 3,3′-diaminobenzidine (Dako) for 30 sec. Tissue was rinsed in dH_2_0, dehydrated, counterstained with hematoxylin and mounted. Negative controls consisted of incubation with a non-specific rabbit IgGs and omission of the primary antibody.

### Statistical analysis

Statistical analyses were performed with Graph Pad Prism v4.0 (GraphPad Software Inc., San Diego, CA, USA). An unpaired Student's t-test, or a Mann Whitney non-parametric test was applied when group variances were not equal or the data was not normally distributed, to compare the means of two groups. A chi-squared test was employed when analyzing proportional data. P-values of *P*<0.05 were regarded as significant.
